# *Artemia* spp., a Susceptible Host and Vector for Lymphocystis Disease Virus

**DOI:** 10.3390/v11060506

**Published:** 2019-06-01

**Authors:** Estefania J. Valverde, Alejandro M. Labella, Juan J. Borrego, Dolores Castro

**Affiliations:** Departamento de Microbiología, Facultad de Ciencias, Universidad de Málaga, 29017 Málaga, Spain; ejv@uma.es (E.J.V.); amlabella@uma.es (A.M.L.); jjborrego@uma.es (J.J.B.)

**Keywords:** Lymphocystis disease virus, *Artemia* spp., viral infection, *Sparus aurata*, viral transmission

## Abstract

Different developmental stages of *Artemia* spp. (metanauplii, juveniles and adults) were bath-challenged with two isolates of the Lymphocystis disease virus (LCDV), namely, LCDV SA25 (belonging to the species *Lymphocystis disease virus 3*) and ATCC VR-342 (an unclassified member of the genus *Lymphocystivirus*). Viral quantification and gene expression were analyzed by qPCR at different times post-inoculation (pi). In addition, infectious titres were determined at 8 dpi by integrated cell culture (ICC)-RT-PCR, an assay that detects viral mRNA in inoculated cell cultures. In LCDV-challenged *Artemia,* the viral load increased by 2–3 orders of magnitude (depending on developmental stage and viral isolate) during the first 8–12 dpi, with viral titres up to 2.3 × 10^2^ Most Probable Number of Infectious Units (MPNIU)/mg. Viral transcripts were detected in the infected *Artemia*, relative expression values showed a similar temporal evolution in the different experimental groups. Moreover, gilthead seabream (*Sparus aurata*) fingerlings were challenged by feeding on LCDV-infected metanauplii. Although no Lymphocystis symptoms were observed in the fish, the number of viral DNA copies was significantly higher at the end of the experimental trial and major capsid protein (*mcp*) gene expression was consistently detected. The results obtained support that LCDV infects *Artemia* spp., establishing an asymptomatic productive infection at least under the experimental conditions tested, and that the infected metanauplii are a vector for LCDV transmission to gilthead seabream.

## 1. Introduction

The family *Iridoviridae* comprises two subfamilies and six genera [1]. Three of them, *Lymphocystivirus*, *Megalocytivirus,* and *Ranavirus* (subfamily *Alphairidovirinae*), infect ectothermic vertebrates (amphibians, reptiles and bony fish), whereas the hosts for the other three genera, *Iridovirus*, *Chloriridovirus*, and *Decapodiridovirus* (subfamily *Betairidovirinae*), are invertebrates (primarily insects and crustaceans) [1,2,3].

Members of the genus *Lymphocystivirus*, collectively named as Lymphocystis disease virus (LCDV), are the causative agents of the Lymphocystis disease (LCD) affecting a wide variety of freshwater, brackish, and marine fish species [4]. The characteristic lesions of LCD are small pearl-like nodules on fish skin and fins, grouped in raspberry-like clusters of tumorous appearance [5,6]. Although this disease is rarely fatal, affected fish cannot be commercialized, provoking important economic losses [7]. LCD is the main viral infection reported in gilthead seabream (*Sparus aurata*) aquaculture [8] and it is caused by *Lymphocystis disease virus 3* (LCDV-Sa). Two more species have been recognized in the genus *Lymphocystivirus*—*Lymphocystis disease virus 1* (LCDV1) and *Lymphocystis disease virus 2* (LCDV-C), that infect European flounder (*Platichthys flesus*) and Japanese flounder (*Paralichthys olivaceus*), respectively—and a number of isolates have been obtained from other LC-diseased fish species, but their taxonomic position is unclear [1].

It is assumed that LCDV transmission occurs through the skin and gills of fish by direct contact or by waterborne exposure [9,10]. However, viral transmission via the alimentary canal has been demonstrated for gilthead seabream larvae feeding on LCDV-positive rotifers [11].

The brine shrimp *Artemia* (Crustacea, Branchiopoda, Anostraca) is an aquatic crustacean frequently used for the feeding of postlarvae fish in aquaculture practice [12,13]. Several authors have considered *Artemia* spp. nauplii as a possible source for the introduction of microorganisms into the rearing systems, including bacteria, viruses, and protozoa [14,15,16,17,18,19], and in most studies, a mechanical carrier stage, in which the pathogen does not multiplicate into the brine shrimp but accumulates in its alimentary canal, has been proposed [16,19,20].

LCDV has been detected by PCR-based methods in *Artemia* cysts and nauplii/metanauplii collected in gilthead seabream hatcheries [21,22]. In a previous study, we demonstrated that the nauplii become easily contaminated with LCDV by immersion challenge. Furthermore, infectious LCDV persists along the *Artemia* life cycle, with viral genome and antigens detected not only in the gut of adult specimens, which could be related to a viral bioaccumulation process, but also in the ovisac in females [21]. These findings suggest that *Artemia* might act as a reservoir of LCDV and could support viral replication.

The aim of the present study was to investigate the susceptibility of different developmental stages of *Artemia* spp. to LCDV and to elucidate its role as a vector for LCDV transmission to gilthead seabream.

## 2. Materials and Methods

### 2.1. Brine Shrimp Culture

*Artemia* spp. cysts (Artemia AF, INVE Aquaculture Inc., Salt Lake City, UT, USA) were decapsulated using a mixture of sodium hypochlorite (0.5 g active chlorine per gram of cysts) and sodium hydroxide (0.15 g/g cysts), following a standard procedure [23]. Residual hypochlorite was neutralized with sodium thiosulfate (0.1%, *w*/*v*, 5 min). Decapsulated cysts were hatched in sterile seawater (33 g/L salinity) at 26 °C [12]. After a 48 h incubation, hatched instar II nauplii were separated from the unhatched and empty cysts and transferred to aquaria with fresh sterile seawater. Nauplii were reared to the adult stage at 26 °C, with continuous aeration and a 24 h photoperiod, and fed with a commercial phytoplankton-based food (Mikrozell-Hobby, Dohse Aquaristik GmbH, Grafschaft-Gelsdorf, Germany). Different developmental stages (nauplii, metanauplii, juveniles and adults) were taken from this stock at 4, 8, 14, and 21 d post-hatching (dph), respectively, and used in the experimental infections described below.

### 2.2. LCDV Infection in Brine Shrimp

The infectivity of LCDV to different developmental stages of *Artemia* (metanauplii, juveniles and adults) was tested by immersion challenge, using an inoculum of 10^2^ TCID_50_/mL during 24 h as specified by Cano et al. [21]. After the challenge, animals were filtered through a synthetic net, washed three times for 5 min each in sterile seawater, transferred to aquaria with fresh sterile seawater, and maintained as specified above.

Two LCDV isolates were used for the challenges—LCDV SA25 from gilthead seabream (belonging to genotype VII and identified as LCDV-Sa) [24,25], and LCDV strain Leetown NFH (ATCC VR-342; genotype VIII). Brine shrimps at the same developmental stages inoculated with Leibovitz’s L-15 medium (Gibco, Life Technologies, Carlsbad, CA, USA) were used as control groups.

Pooled samples of brine shrimp, approximately 100 mg in weight, were collected from each experimental group at several times post-inoculation (pi) (1, 3, 5, 8, 12, 15, and 23 dpi). The animals were washed with sterile seawater as specified above, gently dried on sterile filter paper, and frozen in liquid nitrogen. Samples were ground in liquid nitrogen using a Mixer Mill MM400 (Retsch GmbH, Haan, Germany), and subsequently used for both nucleic acid extraction and virological analysis.

### 2.3. Gilthead Seabream Challenge with LCDV-Infected Artemia

Gilthead seabream fingerlings (0.5–1 g) were obtained from a research marine aquaculture facility with no record of LCD. Prior to the experiment, 10 fish were randomly collected and analyzed by real-time PCR (qPCR) [22] to ensure that they were LCVD-free. The fish were divided into two groups (50 individuals per group) and stocked at a density of 2 g/L in aquaria with filtered seawater. Fish were maintained at 20–22 °C and a 12-h photoperiod, and fed with commercial pellets (Gemma PG 0.8, Skreeting, Burgos, Spain) at a feeding rate of approximately 5% fish body weight per day.

*Artemia* nauplii were inoculated by immersion with LCDV isolate SA25 or Leibovitz’s L-15 medium as previously specified. At 8 dpi, metanauplii were washed with sterile seawater and used to feed gilthead seabream fingerlings. The presence of LCDV in brine shrimp (two samples of 100 mg per experimental group) was determined by qPCR following the procedure described in Section 2.6.

For the oral challenge, fingerlings were fed once with metanauplii that had been inoculated with LCDV or L-15 medium (challenged and control groups, respectively) at a concentration of 0.2 g/L. The following day, the commercial diet was resumed, and fish were maintained at the conditions indicated above for 30 d. Fingerlings were euthanized by anaesthetic overdose (150 mg/mL MS-222, Sigma-Aldrich, St. Louis, MO, USA). All procedures were carried out following the European Union guidelines for the protection of animals used for scientific purposes (Directive 2010/63/UE).

Seven gilthead seabream fingerlings from both the oral challenged and control groups were randomly sampled at 7, 12, and 24 dpi. Samples, consisting of the caudal part of the body (approximately the posterior one third of the fish body), were homogenized in L-15 medium (10%, *w*/*v*) [26] and used for nucleic acid extraction.

### 2.4. Virological Analysis

A total of 50 mg of brine shrimp tissue powder was suspended in 1 mL of Leibovitz’s L-15 medium supplemented with 2% l-glutamine, 1% penicillin-streptomycin and 2% foetal bovine serum, and clarified by centrifugation (10,000× *g* for 5 min at 4 °C) (Rotina 38R centrifuge, Hettich, Kirchlengern, Germany). These homogenates were used to inoculate SAF-1 cells [27], BF-2 cells for homogenates from LCDV ATCC VR-342 infected animals, or were kept at −20 °C until used for virus titration. Cell cultures were maintained at 20 °C until the appearance of cytopathic effects (CPE) (up to 14 dpi). Infectious titres were determined in SAF-1 or BF-2 cells using the ICC-RT-PCR assay described by Valverde et al. [25]. Briefly, cells were inoculated in triplicate with ten-fold serial dilutions of the homogenates and harvested at 5 dpi for total RNA extraction using a commercial kit. After DNase I treatment, one step RT-PCR was performed using primers targeting the major capsid protein (*mcp*) gene. Amplification products were denatured and detected by blot-hybridization using a specific DNA probe. Viral titre, expressed as MPNIU/mL, was estimated using an MPN table with a confidence level of 95%.

### 2.5. DNA and RNA Extraction and cDNA Synthesis

Total DNA and RNA were extracted from 20 mg of tissue powder (brine shrimp samples) or 200 μL of homogenate (fish samples) using the Illustra triplePrep Kit (GE Healthcare, Chicago, IL, USA), following the manufacturer’s instructions. Total RNA was treated with RNase-free DNase I (Sigma-Aldrich) for 30 min at 37 °C. RNA purity and quantity were determined using a NanoDrop 1000 (Thermo Scientific, West Palm Beach, FL, USA). After DNase treatment, total RNA was used in the qPCR reaction in order to control for the absence of viral genomic DNA. First-strand DNA synthesis was carried out with 1 μg of total RNA and random hexamer primers using the Transcriptor First Strand cDNA Synthesis Kit (Roche Life Science, Indianapolis, IN, USA). DNA and cDNA were stored at −20 °C until used as template for qPCR.

### 2.6. LCDV DNA Quantification and Gene Expression

Viral DNA quantification was carried out by qPCR using the methodology described by Valverde et al. [22]. Viral loads were expressed as copies of viral DNA per milligram of tissue.

The *mcp* gene expression was analyzed as an indicator of viral productive infection. Relative quantification of *mcp* gene expression was carried out by RT-qPCR, following the protocol mentioned above but using 20 μL final volume reactions and cDNA generated from 200 ng of the original RNA template.

For LCDV-challenged *Artemia*, relative viral gene expression values were calculated using the comparative delta-Ct method with *Artemia actin* expression used for normalization. Primers for *Artemia actin* gene detection by qPCR (Art-actin-F: 5′-GGTCGTGACTTGACGGACTATCT-3′, and Art-actin-R: 5′- AGCGGTTGCATTTCTTGTT-3′) were designed using Primer Express Software v3.0 (Applied Biosystems, Life Technologies, Carlsbad, CA, USA) based on the sequence obtained from GenBank (accession no. X52602.1). No significant differences in Ct values were observed for this housekeeping gene between different experimental groups during the course of the infection (CV = 1.02%, Kruskal-Wallis test H = 2.27, *p* = 0.13).

In the case of gilthead seabream fingerlings challenged by feeding, normalized relative *mcp* expression levels were calculated by applying the formula F = log_10_ [(E + 1)^40−Ct^/N] [28], where E is the amplification efficiency of the qPCR, Ct (threshold cycle) corresponds to the PCR cycle number, N is the maximal number of viral DNA copies/mg of tissue detected minus the number of viral DNA copies/mg of tissue determined by absolute qPCR for the sample, and Ct of 40 arbitrarily corresponds to “no Ct” by qPCR. In this challenge, results obtained for viral DNA quantification and relative gene expression were analyzed using a Mann–Whitney U test followed by a Holm–Bonferroni correction for multiple comparisons.

## 3. Results

### 3.1. Infection of Artemia spp. by LCDV

**Figure 1:**
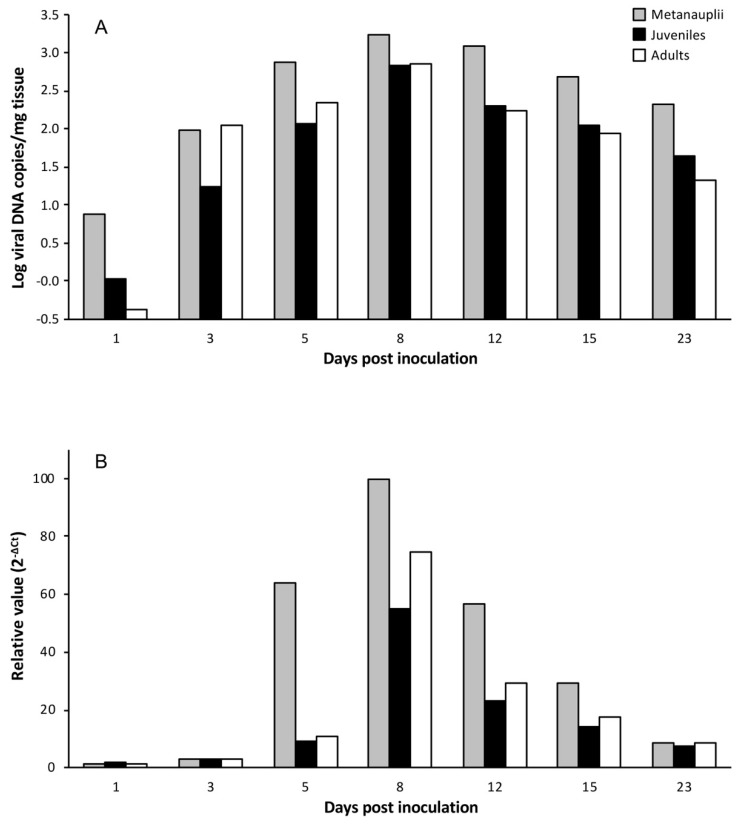
Temporal evolution of viral loads (**A**) and relative major capsid protein (*mcp*) gene expression values (**B**) in different developmental stages of *Artemia* inoculated with Lymphocystis disease virus (LCDV) SA25.

To establish if LCDV replicates in *Artemia* spp. cells, experimental infections were carried out using LCDV SA25 and three developmental stages of *Artemia*. The time course of the experimental infection was studied by analyzing viral load and *mcp* gene expression in parallel. In challenged metanauplii, the viral load increased by more than two orders of magnitude from the first to the 8th dpi (from 7.6 × 10^0^ to 1.7 × 10^3^ copies of viral DNA/mg of tissue). The viral load remained above 10^2^ copies of viral DNA/mg during the entire sampling period (Figure 1A). In juveniles and adults, the time course of the infection was similar to that obtained for metanauplii, reaching the maximal value at 8 dpi (6.7 × 10^2^ and 7 × 10^2^ copies of viral DNA/mg of tissue, respectively) (Figure 1A). Relative expression of viral *mcp* transcripts showed a similar temporal evolution for the three experimental groups analyzed, reaching the highest value at 8 dpi (Figure 1B). Neither LCDV genomes nor mRNA were detected in brine shrimp inoculated with L-15 medium (control groups).

No CPE could be observed in cell cultures inoculated with LCDV-infected *Artemia* homogenates and maintained up to 14 dpi. Nevertheless, by using the ICC-RT-PCR assay, viral infectious titre determination was carried out at 8 dpi. The estimated viral titres were 9.3 × 10^1^ MPNIU/mg for metanauplii and juveniles, and 2.3 × 10^2^ MPNIU/mg for infected adults.

**Figure 2:**
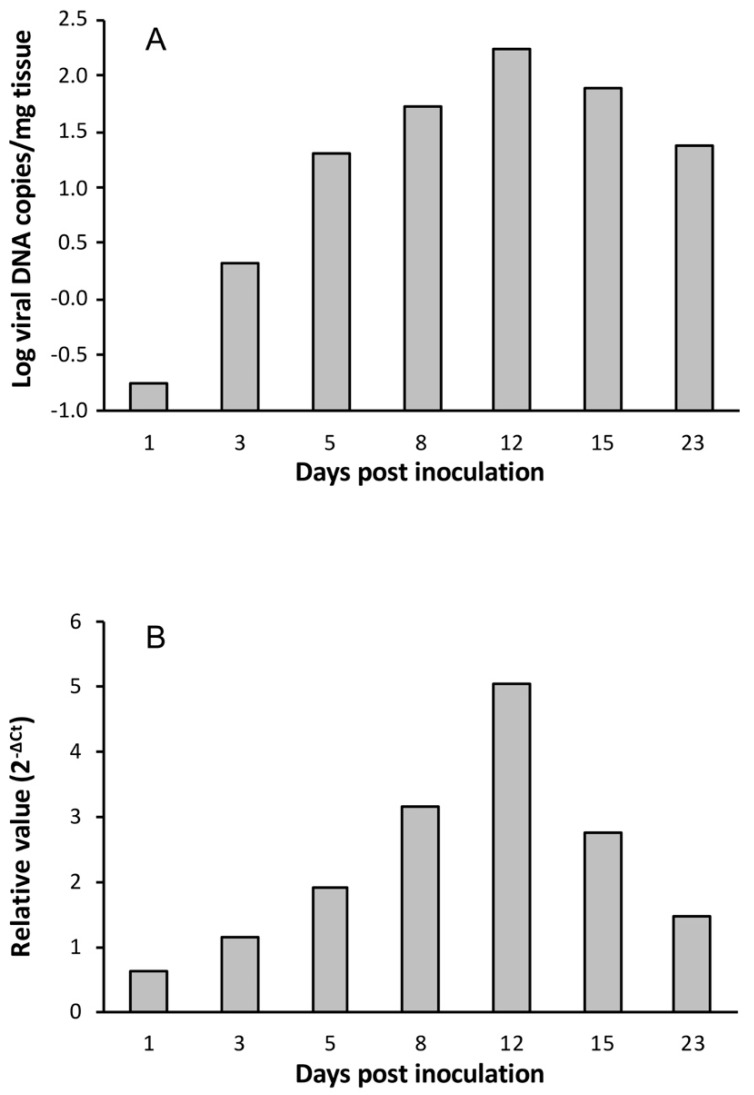
Temporal evolution of viral loads (**A**) and relative *mcp* gene expression values (**B**) in *Artemia* metanauplii inoculated with LCDV ATCC VR-342.

Viral load and *mcp* gene expression were also investigated in *Artemia* metanauplii challenged with LCDV ATCC VR-342. Viral load reached the maximal value at 12 dpi (1.7 × 10^2^ copies of viral DNA/mg of tissue), and the same was observed for relative viral gene expression (Figure 2). In this case, the viral titre at 8 dpi was 7.5 MPNIU/mg, one order of magnitude lower than obtained in metanauplii infected by LCDV SA25.

In any of the experimental groups, clinical signs or mortality were not observed in the *Artemia* cultures.

### 3.2. LCDV Transmission to Gilthead Seabream Fingerlings

*Artemia* metanauplii used for fingerlings feeding were infected by LCDV, as demonstrated by qPCR, with an estimated viral load of 1.2 ± (0.1) × 10^3^ copies of viral DNA/mg of tissue, whereas metanauplii in the control group remained LCDV-negative.

**Figure 3:**
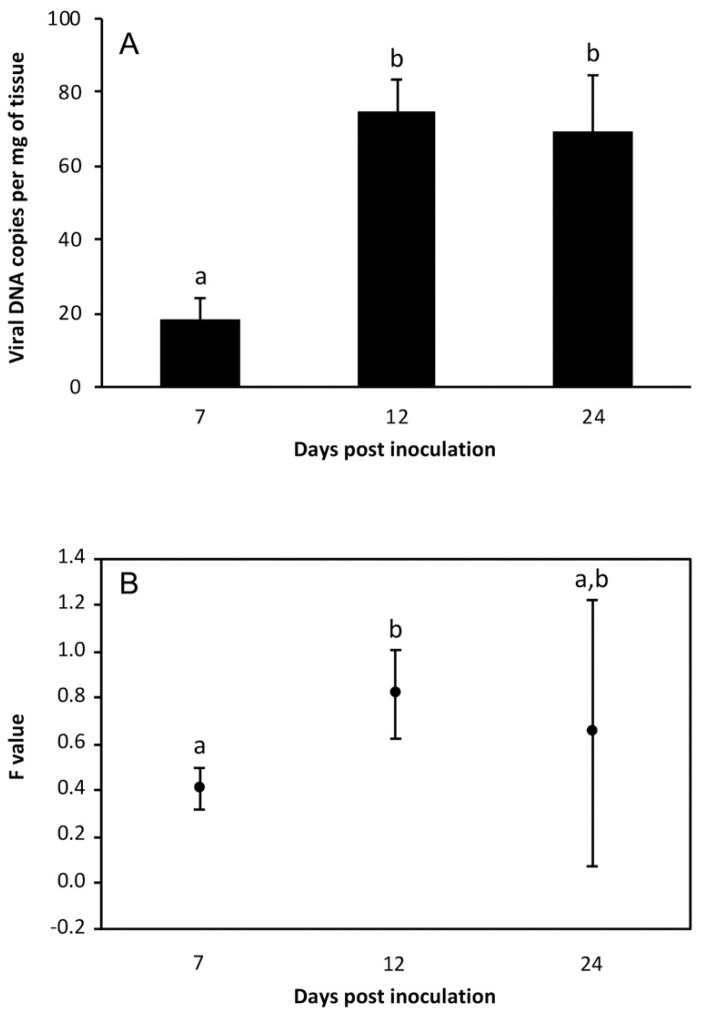
Viral loads (**A**) and relative *mcp* gene expression values (**B**) in gilthead seabream fingerlings orally challenged with LCDV-positive *Artemia* metanauplii (mean ± standard deviation; *n* = 7). Different letters indicate significant differences (*p* < 0.01) (Mann–Whitney U-test, Holm–Bonferroni correction).

In fingerlings fed on the LCDV-positive metanauplii (challenged group), LCDV was detected by qPCR in all fish and at all time points analyzed. At 7 dpi, the estimated viral load ranged between 10.6 and 26.8 copies of viral DNA per mg of tissue. Five days later, viral loads were significantly higher (*p* < 0.01), and they remained at similar values at 24 dpi (Figure 2A). No LCD symptoms were observed in these fish at the end of the experiment (30 dpi). The *mcp* gene expression was also detected in all fish analyzed, with the highest F-value observed at 12 dpi (Figure 3B). Neither LCDV genomes nor mRNA were detected in fish from the control group (i.e., fed on metanauplii inoculated with L-15 medium).

## 4. Discussion

A number of studies have confirmed the role of *Artemia* nauplii as vectors for several crustacean viruses, such as *Macrobrachium rosenbergii* nodavirus (MrNV), hepatopancreatic parvo-like virus (HPV), white spot syndrome virus (WSSV), and infectious myonecrosis virus (IMNV) [29,30,31,32]. In addition, *Artemia* appears to be susceptible to some of these viruses, including WSSV and MrNV, with the infection being asymptomatic [33,34]. Regarding fish pathogens, *Artemia* nauplii have proven to be a mechanical vector only in the case of microsporidia and *Vibrio anguillarum* [35,36], although some studies have shown that they could also accumulate viral pathogens and protozoa [16,19,37].

Previous studies demonstrated that infectious virus could be detected in *Artemia* nauplii inoculated with LCDV-Sa by immersion and the virus persisted to the adult stage, and from adults to reproductive cysts [21]. These results led us to consider the hypothesis that *Artemia* spp. could be susceptible to LCDV infection, acting as reservoir and biological vector for LCDV.

The results obtained in the experimental infections carried out demonstrated that *Artemia* spp. could be infected by LCDV at different developmental stages, since viral loads increased during the course of the experiments. In addition, viral transcripts were also detected, showing a similar temporal evolution. Thus, *Artemia* spp. seems to be a susceptible host for LCDV, at least in experimental conditions, with the resulting infection being asymptomatic. This is the first description of a fish virus that also infects invertebrates. Viral loads and infectious titres estimated in LCDV-infected *Artemia* were higher than those previously obtained for subclinically infected gilthead seabream fingerlings or juveniles [25,28,38].

During the course of the experimental infections, particularly in those performed with metanauplii and juveniles, brine shrimps kept growing, doubling or tripling in size, and completed their life cycle, becoming adults. Thus, viral loads expressed per mg of tissue are difficult to interpret, and do not reflect actual viral loads per individual. Taking this into account, the number of genome copies probably increased in each brine shrimp specimen during the experimental trial.

Viral replication kinetics were similar in the experimental infections carried out with different developmental stages of *Artemia*. Nevertheless, relative viral gene expression values were higher in metanauplii compared to juveniles or adults infected by LCDV SA25, although this difference was not reflected in infectious titres. Differences in relative viral expression values were observed in metanauplii infected by both viral isolates, which might indicate that viral infectivity is variable among LCDV genotypes or that *Artemia* susceptibility to these isolates differs.

On the other hand, gilthead seabream fingerlings fed on LCDV-infected metanauplii became infected, as demonstrated by the increase in the number of LCDV DNA copies during the experimental period, and the detection of viral transcripts in these animals. Estimated viral loads were consistent with those previously reported for asymptomatic infections in gilthead seabream [25]. These results indicate that *Artemia* metanauplii can be a vector for LCDV, participating in viral transmission to gilthead seabream via the alimentary route under laboratory conditions that mimics those used in aquaculture farms. Whether *Artemia* is also a reservoir host for LCDV remains to be investigated.

In conclusion, the results demonstrate that LCDV establishes a productive infection in *Artemia* spp., at least under the experimental conditions tested, which extend the host range of the genus *Lymphocystivirus* to crustaceans. Furthermore, our study confirms that *Artemia* metanauplii act as a vector for LCDV transmission to gilthead seabream fingerlings.
